# Delivering maternal mental health through peer volunteers: a 5-year report

**DOI:** 10.1186/s13033-019-0318-3

**Published:** 2019-09-17

**Authors:** Najia Atif, Amina Bibi, Anum Nisar, Shaffaq Zulfiqar, Ikhlaq Ahmed, Katherine LeMasters, Ashley Hagaman, Siham Sikander, Joanna Maselko, Atif Rahman

**Affiliations:** 1 Human Development Research Foundation, Mandra, Gujar Khan, Pakistan; 2 University of North Carolina at Chapel Hill, Chapel Hill, NC USA; 3 Health Services Academy, Opp. NIH, Chak Shahzad, Islamabad, Pakistan; 4 Department of Psychological Sciences, Institute of Life and Human Sciences, University of Liverpool, Block B, Waterhouse Building, 1-5 Dover Street, Liverpool, L69 3BX UK

**Keywords:** Perinatal depression, Maternal depression, Peer volunteers, Psychosocial intervention, Thinking Healthy Programme, Low and middle income countries, Task-shifting, Cognitive behaviour strategies

## Abstract

**Background:**

Maternal depression affects one in five women in low-and middle income countries (LMIC) and has significant economic and social impacts. Evidence-based psychosocial interventions delivered by non-specialist health workers are recommended as first-line management of the condition, and recent studies on such interventions from LMIC show promising results. However, lack of human resource to deliver the interventions is a major bottle-neck to scale-up, and much research attention has been devoted to ‘task-sharing’ initiatives. A *peer*-*delivered* version of the World Health Organization’s Thinking Healthy Programme for perinatal depression in Pakistan and India showed clinical, functional and social benefits to women at 3 months postpartum. The programme has been iteratively adapted and continually delivered for 5 years in Pakistan. In this report, we describe the extended intervention and factors contributing to the peers’ continued motivation and retention, and suggest future directions to address scale-up challenges.

**Methods:**

The study was conducted in rural Rawalpindi. We used mixed methods to evaluate the programme 5 years since its initiation. The competency of the peers in delivering the intervention was evaluated using a specially developed Quality and Competency Checklist, an observational tool used by trainers to rate a group session on key areas of competencies. In-depth interviews explored factors contributing to the peer volunteers’ continued motivation and retention, as well as the key challenges faced.

**Results:**

Our key findings are that about 70% of the peer volunteers inducted 5 years ago continued to be part of the programme, retaining their competency in delivering the intervention, with only token financial incentives. Factors contributing to sustained motivation included altruistic aspirations, enhanced social standing in the community, personal benefits to their own mental health, and the possibility for other avenues of employment. Long-term challenges included demotivation due to lack of certainty about the programme’s future, increased requirement for financial incentivisation, the logistics of organising groups in the community, and resistance from some families to the need for ongoing care.

**Conclusions:**

The programme, given the sustained motivation and competence of peer volunteers in delivering the intervention, has the potential for long-term sustainability in under-resourced settings and a candidate for scale-up.

## Introduction

Maternal mental health is an important public health priority. Maternal depression, the mental health condition with the greatest public health impact, affects approximately one in five women in low and middle income countries (LMIC). Children of mothers with depression are 1.5 times more likely to be underweight and 1.4 times more likely to be stunted [1]. In high income countries (HIC), the value of total lifetime costs of maternal depression has been estimated to be over USD $100,000 per woman with the condition, with the majority of the costs related to adverse impacts on children [2]. In LMIC where maternal depression affects more women and is independently associated with infant malnutrition, the relative impact is likely to be greater.

The Thinking Healthy Programme (THP) is an evidence-based psychosocial intervention recommended by the World Health Organization (WHO) as the first-line management of perinatal depression in primary and secondary care settings [3]. THP uses cognitive behaviour therapy techniques, including behavioural activation and problem solving, integrated into the routine work of community health workers (CHWs). The intervention consists of 16 sessions delivered during pregnancy and 6–8 months postnatal, focusing on the mother’s well-being and its effect on her relationship with the infant and significant others. The intervention was first tested in Pakistan which has a well-established publicly funded Community Healthy Worker programme called the “Lady Health Workers” (LHW) programme. Lady Health Workers live in the same villages they serve, being responsible for providing preventive and promotive health care to about 120 households in their neighbourhood. A large randomised controlled trial (RCT) showed that THP more than halved the rate of depression compared with usual care and led to significant improvements in women’s functioning and disability [3]. Importantly, benefits were also observed on infant outcomes: diarrhoeal rates were reduced and immunization rates were increased, and the intervention was effective in the poorest populations. Drawing on this evidence, the Thinking Healthy manual for perinatal depression was incorporated into the WHO’s flagship Mental Health Gap Action Programme (mhGAP) and is available at the WHO’s website http://www.who.int/mental_health/maternal-child/thinking_healthy/en/.

Despite these efforts, the majority of women with perinatal depression, especially in areas with the least resources, do not receive the treatment they need. Key reasons include shortage of community health workers in overstretched primary care systems, and competing priorities leading to excessive workload of existing CHWs. To address this bottle-neck we conducted further studies in two countries, Pakistan and India, to evaluate the effectiveness of THP when delivered by *peer volunteers* (women from the same villages with no prior experience of health-care delivery but sharing the same culture, socioeconomic status and life-experiences as their clientele). The core elements of the Thinking Healthy Programme including developing an empathetic relationship, facilitating family support, behavioral activation and problem-solving were retained but simplified through the use of vignettes, culturally adapted pictures, and everyday terms to describe distress [4]. The intervention was delivered during pregnancy and up to 6 months postnatal. In Pakistan, the peer volunteers worked alongside the established Lady Health Workers, who give them credibility and ease of access to the households under their care. The Thinking Healthy Programme-Peer-delivered (THPP) manual is available at http://hdrfoundation.org/publications (in “Training Materials”) (last accessed 12 July 2019).

Randomised controlled trials from Pakistan and India showed that THPP led to improved clinical, social, and functional outcomes in women compared to enhanced usual care at 3 months postnatal [5, 6]. This research showed that the volunteer peers working alongside CHWs had the potential to overcome the key human resource barriers to providing an accessible and culturally acceptable intervention to women living in the most under-served areas of the world. As encouraging as these findings are, it is important to evaluate the long-term sustainability of such programmes.

In one of the sites (Pakistan), the THP-P trial has continued to evaluate the long-term impact of the programme [7]. In addition to clinical outcomes (to be reported on completion of an ongoing RCT), it would be important to examine the long-term sustainability of the intervention-delivery process through peer volunteers. In this report, we describe the extended peer-delivered intervention and the peers’ long-term competence in delivering it, their motivation and retention to the programme, challenges, and future directions of the programme, 5 years after its inception.

## Methods

### The settings and context

The study was conducted in Kallar Syeddan (population 400,000), a sub-district of Rawalpindi in the province of Punjab, Pakistan. The sub-district is typical of a socioeconomically deprived rural area in a low-income country, with high rates of poverty (up to 25% on less than US$3.5 per day), high fertility rates (3.8) and low levels of female education (less than 45% literacy rate). The area is largely agrarian, consisting of close-knit communities living in villages and large household sizes (6.2 persons per household) including extended and joint families (consisting of multiple generations; parents, their children, and the children’s spouses and offspring, living in the same house). Women are generally economically and socially dependent on significant members of their families (husband, mother-in-law, own parents). The publicly-funded primary health care system consists of a Basic Health Unit covering a population of about 30,000 and served by a doctor, trained midwife, 2–3 paramedics and 15–20 village-based community health workers (Lady Health Workers), once semi-volunteers and but now regular government employees, who deliver largely preventive services and health promotion in the community. Epidemiological studies from the study area indicate rates of perinatal depression between 26 and 33%. The peer-delivered Thinking Healthy Programme was initiated in July 2014. The 6-months evaluation of the programme has been reported elsewhere [5] and the 36-month evaluation is currently underway [7].

### The ‘extended’ peer-delivered Thinking Healthy Programme

Taking advantage of the peers’ continued presence in the community and willingness to remain engaged with the mothers with whom they had developed an empathetic relationship, the Thinking Healthy sessions were extended to continue through the first 1000 days of the child’s life instead of ending at 6 months. The rationale for the extended intervention was two-fold: First, depression is a chronic and relapsing condition, especially in populations living in socioeconomically adverse environments. In the absence of mental health facilities offering regular follow-up, it would be important for peer volunteers to continue engaging with the mothers so they could benefit from the ongoing social support offered. Second, the first 1000 days of the newborn’s life are characterized by rapid rates of neuronal proliferation, growth and differentiation, myelination, and connectivity. This period provides the best opportunity to ensure an optimal environment for normal development. Peer volunteers could use their established relationship with the mothers and significant family members to provide education to the families to best enhance this development.

The extended intervention began after the child was 6 months old and consisted of 18 group ‘booster’ sessions which were delivered monthly for 6 months, and subsequently every 2 months, until the child was 3 years old. These group sessions provided a safe environment to women to voice their problems, share experiences of childcare, and provide mutual support. The peer volunteers were trained to use culturally-grounded vignettes that served as tools to deliver health and well-being messages. Derived from our qualitative studies, these vignettes depicted a variety of real-life challenges and situations faced by rural women with young children. They aimed to help participants reflect and gain better insight about their own problems and allowed them to share personal experiences of overcoming similar problems. In addition to the narratives, card games containing pictorial illustrations of personal well-being messages were developed. These aimed to challenge unhelpful thinking patterns and behaviour and to replace them with alternative thoughts and behaviour in a fun and interesting way. The sessions aimed to enhance not just maternal well-being but also child-care and development by encouraging mother-infant interaction and play. The intervention provided examples of age-appropriate activities, derived from the WHO’s Care for Development Package [8] and encouraged demonstration of these activities during the sessions. While the ‘booster’ group sessions did not focus on specific strategies to address depression, the peer could still draw on her knowledge and skills of specific psychotherapeutic elements such as behavioural activation when required. A reference manual of the extended THP-P is available at http://hdrfoundation.org/publications (under “Training Materials”) (last accessed 12 July 2019).

#### Selection, training and supervision of peer volunteers

The original 45 peer volunteers inducted in July 2014 had been identified with the help of the Lady Health Workers who had intimate knowledge of the community. Peer volunteers were recruited with the following criteria in mind: local residence, women of child-bearing age, similar life circumstances to participants’ (e.g., socioeconomic status or having experienced perinatal depression), good communication skills, and a favorable reputation in the community. The THPP training is described in detail elsewhere [9]. Briefly, the THPP programme adopted a cascade model of training and supervision—the peer volunteers were trained and supervised by non-specialist THPP facilitators, who in turn were trained and supervised by a specialist. The peers’ training in the extended intervention employed the same cascade model, and consisted of 4 days of classroom and 2 days of field training, on top of the original 5-day of classroom training and 3 months of field training they had received [9]. The extended training focused on group facilitation skills, using culturally appropriate stories and accompanying pictorial illustrations to help mothers gain deeper understanding of their issues, and facilitating the sharing of experiences, mutual support and problem solving. The training also provided basic knowledge of child-development at various stages to enable them to deliver appropriate health messages. They were provided monthly group and field supervisions to provide ongoing support and experiential learning and to ensure their motivation and fidelity to the intervention.

### Evaluation procedures

The programme was evaluated 5 years after initiation using mixed methods. The competency and skills of the peer volunteers were evaluated using a specially developed Quality and Competency Checklist, an observational tool used by trainers to rate a group session on 6 areas of competencies, namely the peers’ ability to: (1) develop an empathetic relationship, (2) encourage family support and participation, (3) deliver the contents of the group sessions, (4) generate problem-solving strategies, (5) facilitate group discussions and (6) deal with challenging situations. The items in each area were rated on a Likert scale (0–2) ranging from “not demonstrated” to “partially demonstrated” and “demonstrated well”, with an option of not applicable. Each area was separately scored and the score was converted into percentage. A minimum score of 70% in each area indicated satisfactory competency.

This study used in-depth interviews to gain a thorough understanding of peers’ experiences of delivering the intervention to the community. The in-depth interviews explored factors contributing to the peer volunteers’ continued motivation and retention, as well as the key challenges faced. Data were collected till saturation was achieved. All interviews were recorded, transcribed verbatim and analysed using the Framework Analysis [10]. The Framework Analysis allows the data to be analysed systematically using in five stages: familiarisation, development of the thematic framework or index, indexing, charting and interpreting the data. Researchers worked in pairs to familiarize themselves with and code the data. Codes were clustered based on their similarities and differences to form themes and subthemes, leading to the development of a thematic framework. The process was overseen by the lead-author (NA) and any discrepancies in the findings were discussed and resolved through discussion, revisiting the raw data and referring to the field notes. During this process, the raw data were revisited and indexed, in order to revise and finalize the Thematic Framework. Following this, charts were developed that involved summarising the indexed data and placing them under the appropriate theme in the chart. All summaries included in the charts were referenced to allow an audit trail of the findings. Finally, we synthesised and interpreted elements of the charts and interpreted and highlighted key findings.

## Results

### Competency of peer volunteers’ skills and knowledge 5 years after programme implementation

Of the cohort of 45 peer volunteers initially recruited 31 were retained over 5 years and showed sustained or improved competencies over time, with all 31 achieving satisfactory competence. Of the peer volunteers who dropped out over 5 years, 6 (13%) did so because they were unable to achieve or maintain the required level of competency. This demonstrated the success of the cascaded model of training and supervision [9], and also indicated that the intervention was being delivered with sustained fidelity across the 5 year period.

### Retention and motivation of peer volunteers 5 years after programme implementation

As described above, 31/45 (70%) of the peer volunteers initially recruited were retained over the 5 year period. Most peer volunteers who left the programme did so in the first year. They were able to identify a replacement for themselves, thus retaining the numbers needed to run the programme smoothly. The reasons for leaving were inability to achieve competence (n = 6), changes in their life circumstances (n = 5), or moving out of the study area (n = 3).

### Factors contributing to sustained engagement and motivation

Several themes emerged from the in-depth interviews of the peers (n = 15) which are summarised below, along with relevant quotations where necessary. These emerged from triangulation of data from in-depth interviews and focus-group discussions with the peer volunteers, and detailed supervision notes kept by the supervisors, through the course of the programme.

#### Altruism and perceived impact on community

The peer volunteers saw their role as altruistic and contributing to the betterment of their community. Delivering the intervention over the extended period gave the peer volunteers an opportunity to directly observe and appreciate the improvements in the mothers’ wellbeing, which they found rewarding.

*“What could be more rewarding than to see a mother smiling again and playing joyfully with her baby. I feel proud of my work as it is bringing positive changes in the lives of many mothers.”*

#### Opportunity for expanding social networks

Most women in this conservative rural setting had little opportunity to socialise outside their close families. Working with the programme gave them the opportunity to interact with other mothers and families for an extended period and, in many cases, develop good relationships with them. This befriending also contributed to engagement with the families.

*“Mothers and their families are nice to me. I have made so many friends with the families I work. The mothers, their mothers in*-*law, and sisters in*-*law, they all come to attend the group, which I find very rewarding.”*

Peer volunteers also valued the friendship with other peer volunteers working in their catchment area.

*“I am not short of friends now, every month after our supervision we (peer volunteers) spend some time talking to each other and sometime visit each other’s homes, if in neighbourhood.”*

#### Opportunity for self-improvement

The intervention served as a self-help guide for many peer volunteers and helped with their own mental health issues, as well as issues of child care. They were able to apply the positive problem-solving strategies to their own work. Coupled with the supportive supervision which included positive reinforcement and encouragement, this contributed to improved self-esteem and confidence in many cases.

*“Honestly speaking when I started doing this volunteer work, I never thought it will benefit my own health. I always thought it’s just a job, but after practically doing it I have realized that I have picked up many positive things. I have managed to overcome my own inhibitions and fears. I am confident enough to talk in front of people now.”*

*“I have learnt how important the mother*–*child relationship is. I am a mother of 3 young children and I did not use to pay much attention them. Now that I know its importance I take my time out for them. This is why they are now more attached to me and can talk to me.”*

#### Opportunity for upward mobility

Related to the above, many peer volunteers developed new skills and confidence allowing them to explore new avenues of employment. Two peer volunteers were elected as village councillors to represent other women in their local community. The others found jobs as teaching assistant and community health worker. Of the 5 peer volunteers who left the programme, at least 3 found paid employment outside of their homes for the first time.

*“I got elected as a Lady Councillor to represent my village in the community. This was possible because I met many families working as a volunteer. Was able to help them through difficult situations and this gave me the confidence to extend my role. I am very happy with my new role.”*

#### Financial and ‘in-kind’ incentives

The peer volunteers received a small honorarium of about USD10 per month which included the cost for refreshments during the group session. While this was a very small amount, it still served as an incentive for some of the peer volunteers.

*“The amount of allowance that we get was also an incentive for me to do this job. It was like an additional bonus. I used to save some to treat my daughters.”*

More important was the ‘social investment’ that the peer volunteers made in this close-knit community, which could be construed as a currency through which people paid each other. In other words, a peer might expect ‘in-kind’ returns of support from the community in case of need.

#### Supportive supervision from the programme personnel and LHWs

The peer volunteers received on-going practical and emotional support from their supervisors, field coordinators and the LHWs, which was crucial for their sustained motivation. LHWs liaised between the peer volunteers and the families and helped them in providing access to the families. The field coordinator helped with the logistics of setting up the groups and trouble-shooting. In addition to focusing on knowledge and skills, the supervisors gave a lot of positive reinforcement.

*“Supervisions are very helpful. If we are unable to understand something, our supervisors tries to make us understand in every possible way. Their attitude towards us is very friendly and nice.”*

*“We never felt alone in our mission, we had the support of the LHWs and our supervisors but the best was our field coordinator, whenever we have trouble we ring him, no matter what time of the day it is, he always respond and solve our issue in a day or two.”*

### Challenges encountered

Our previous study reported initial barriers faced by the peer volunteers in their work, including having to negotiate this new role with their own family and gatekeepers, stigma associated with depression in the community, and resistance from some families to accept peer volunteers as health providers [8]. We observed that over time, the peer volunteers’ own families and the community became more accepting towards the peer volunteers. Challenges in the longer-term included the following:

#### Uncertainty about the programme’s future

THPP was implemented as part of a research programme with a limited life, which was extended following further research funding for longer follow-up. The uncertainty about the future of the programme towards the end of the funding cycles affected the morale of the workforce.

*“If the programme finishes, we will not have support to continue this work”.*

#### Increased need for financial incentivisation

As the peer volunteers became more self-assured, confident and skilled, there was a gradual demand for greater financial return for their efforts. We felt that after 2–3 years of initial voluntary work, the demand for greater financial incentivisation was wholly justified, and for sustainability, this cost needed to be built into the financial models of future programmes.

*“I feel I am ready and capable to take on a paid job to help even more women in my community”.*

#### The logistics of organising groups in the community

The peer volunteers were assisted by the Lady Health Workers in organising the groups, but the logistics involved a degree of coordination and time which was an additional burden on the peer volunteers. Some peer volunteers felt obliged to visit women who did not turn up for the groups. In some cases, it was hard to control other community-members from turning up at these groups.

#### Resistance from some families to the need for ongoing care

While the majority of the families became more accepting of the peer volunteers over time, about a quarter did not wish to engage over such a prolonged period and dropped out. While some women no longer required care, others had additional psychosocial problems such as interpersonal violence or severer mental health issues that peer volunteers could not deal with effectively. While this was issue was commonly addressed in supervision, still, some peer volunteers felt helpless and demotivated at this perceived rejection.

## Discussion

Our key findings are that about 70% of the peer volunteers inducted 5 years ago continued to be part of the programme, retaining their competency in delivering the intervention, with only token financial incentives. Factors contributing to sustained motivation included altruistic aspirations, enhanced social standing in the community, personal benefits to their own mental health, and the possibility for other avenues of employment. Long-term challenges included demotivation due to lack of certainty about the programme’s future, increased desire for financial incentivisation, the logistics of organising groups in the community, and resistance from some families to the need for ongoing care. These findings indicate that the programme was sustainable over a 5 year period. However, for the programme to run on its own without any external support and be scaled-up at the provincial or national level, we will need to consider some further modifications. These might include the following:

### Technological enhancements to facilitate training, supervision and delivery

Training and supervision of peer volunteers at scale will be key to maintaining peer motivation as well as quality of work. The cascade method of training can be enhanced by the use of technology, as we have demonstrated in a proof of concept study with Lady Health Workers [11]. The Technology Assisted Cascaded Training and Supervision System (TACTS) used a multimedia android-based training Application. Training materials were converted into Urdu narrative scripts. Culturally appropriate real-life characters (Avatars) representing the trainers, trainees, clients and their family members were developed. The avatars were used to demonstrate skills such as effective use of counseling, collaboration with the mothers’ families and setting health-related tasks. The software was designed to be interactive, prompting trainees to enact role-plays, reflect on their learning, and share relevant experiences within the group. A randomized controlled trial in Pakistan showed no significant differences in competence of health workers trained using TACTS and supervised from distance versus those trained and supervised by a specialist face-to-face [11]. We aim to develop similar applications that can assist peer volunteers in the delivery of the intervention. These enhancements will allow a greater number of peer volunteers to be trained and supervised and make it easier for the peer volunteers to deliver the intervention at fidelity, while retaining the human element so essential to psychosocial interventions.

### Establishing a career-path and continued professional development for peer volunteers

Governmental and non-governmental implementation agencies need to adopt the programme and provide long-term organizational and financial support to provide a sense of certainty for the workforce. This should include a pathway for progression and continued professional development for the peer volunteers. For example, in the second year of the programme, five peer volunteers were identified as ‘champions’ based on their competency scores and outstanding feedback from the community. They shadowed the supervisors, and within a few months, were ready to become peer-supervisors. This graduation of peer volunteers through various levels of competency and experience requires a more systematic approach to evaluation, further professional development and eventual certification so that an entire work-force of qualified peer counsellors can be developed who take on varying levels of responsibilities in a scaled-up programme. Such a career-path will further incentivise and motivate peer volunteers and provide sustainability to the programme.

### Increased need for financial incentivisation

As peer volunteers develop more skills and experience and graduate to greater levels of responsibility, this should be matched by increasing financial incentivisation. We strongly believe that the volunteer or semi-volunteer spirit of the programme should be retained, especially in close-knit communities such as rural Pakistan, as it is a facilitatory factor for both peer-motivation and intervention-quality; the empathetic and altruistic nature of peer volunteers, as well as shared life-experiences, enhances psychosocial interventions. Nevertheless, rational small financial incentivisation could add to the programme strength as well as sustainability. In Pakistan, as in many low-income countries, there is a drive to promote women’s employability. THPP offers a stepping-stone towards one possible career pathway for women. We are currently exploring modalities of collaboration with women development agencies to further develop this model, and exploring business models such as social franchising and public–private partnerships.

### Situating the intervention within a collaborative stepped-care programme of care for depression

The peer delivered Thinking Healthy Programme must be conceptualised as one component of a larger stepped-care programme for depression. About a third of women will have chronic depression, often complicated by psychosocial risks such as intimate partner violence or suicidal ideation. They will only partially respond to the peer-delivered intervention. A well-functioning system of risk assessment, referral and specialist support ought to be part of a scaled-up programme for perinatal depression. This is important not only for the women who need such care, but also for peer volunteers to avoid stress and emotional burn-out. Currently, the supervision system is able to pick up such cases and provide the necessary care, but this provision will also need to be scaled-up in tandem.

## Conclusions

A preliminary evaluation of the peer-delivered programme showed beneficial effects on maternal depression, and a 36-month evaluation of the clinical effectiveness on both mothers and children is currently underway. Even on current evidence, we strongly feel this programme, given the sustained motivation and competence of peer volunteers in delivering the intervention, has the potential for long-term sustainability in under-resourced settings and is therefore an excellent candidate for scale-up. Further innovations and modifications, such as technology-assisted training and delivery, ensuring continued professional development and a career-path for peer volunteers, increased financial incentivisation while retaining the volunteer spirit of the programme, and developing the intervention as part of a larger stepped-care programme for maternal mental health, will be important to assist scale-up and sustainability.

## Data Availability

Not applicable

## Abbreviations

CHW: community health worker
HIC: high income countries
LMIC: low and middle income countries
RCT: Randomised Control Trial
THP: Thinking Healthy Programme
THPP: Thinking Healthy Programme Peer Delivered
WHO: World Health Organisation
